# Supplementary material for: The coefficient of determination R-squared is more informative than SMAPE, MAE, MAPE, MSE and RMSE in regression analysis evaluation
Source: PeerJ Comput Sci. 2021 Jul 5;7:e623. doi: 10.7717/peerj-cs.623 (PMC8279135; doi:10.7717/peerj-cs.623)
Supplement: Supplemental Information 1 [file peerj-cs-07-623-s001.pdf]

SUPPLEMENTARY INFORMATION

		R^2	MAE	MSE	SMAPE	RMSE	MAPE
Random Forests	mean	**0.756**	0.149	0.133	1.808	0.361	0.092
Random Forests	s.d.	0.073	0.025	0.041	0.035	0.058	0.017
Linear Regression	mean	**0.535**	0.283	0.260	1.840	0.498	0.197
Linear Regression	s.d.	0.196	0.036	0.134	0.034	0.108	0.018
Decision Tree	mean	**0.423**	0.157	0.311	0.073	0.546	0.080
Decision Tree	s.d.	0.197	0.050	0.120	0.023	0.113	0.032

Table S1. Regression results on the prediction of hepatitis, cirrhosis, and fibrosis from electronic health records, including standard deviations. Mean values and standard deviations out of 100 executions with 80% randomly chosen data elements used for the training set and the remaining 20% used for the test set. We performed the analysis on the Lichtinghagen dataset (Lichtinghagen et al., 2013; Hoffmann et al., 2018) with the methods employed by Chicco and Jurman (2021). R^2: worst value $-\infty$ and best value +1. SMAPE: worst value 2 and best value 0. MAE, RMSE, MAPE, MSE: worst value $+\infty$ and best value 0.

		R^2	MAE	MSE	SMAPE	RMSE	MAPE
Random Forests	mean	**0.865**	0.412	0.512	0.087	0.714	0.094
Random Forests	s.d.	0.017	0.028	0.067	0.006	0.047	0.007
Decision Tree	mean	**0.426**	1.214	2.170	0.326	1.471	0.286
Decision Tree	s.d.	0.064	0.043	0.236	0.012	0.078	0.010
Linear Regression	mean	**0.254**	1.417	2.828	0.296	1.681	0.325
Linear Regression	s.d.	0.030	0.034	0.117	0.007	0.035	0.011

Table S2. Regression results on the prediction of obesity level from electronic health records, including standard deviations. Mean values and standard deviations out of 100 executions with 80% randomly chosen data elements used for the training set and the remaining 20% used for the test set. We performed the analysis on the Palechor dataset (Palechor and De-La-Hoz-Manotas, 2019; De-La-Hoz-Correa et al., 2019) with the methods Linear Regression, Decision Tree, and Random Forests. R^2: worst value $-\infty$ and best value +1. SMAPE: worst value 2 and best value 0. MAE, RMSE, MAPE, MSE: worst value $+\infty$ and best value 0.
